# Pipeasm: a tool for automated large chromosome-scale genome assembly and evaluation

**DOI:** 10.1093/bioadv/vbaf326

**Published:** 2026-01-02

**Authors:** Bruno Marques Silva, Fernanda de Jesus Trindade, Lucas Eduardo Costa Canesin, Giordano Souza, Alexandre Aleixo, Gisele Nunes, Renato Renison Moreira-Oliveira

**Affiliations:** Environmental Genomics, Instituto Tecnológico Vale, Belém, Pará 66055-090, Brazil; Environmental Genomics, Instituto Tecnológico Vale, Belém, Pará 66055-090, Brazil; Environmental Genomics, Instituto Tecnológico Vale, Belém, Pará 66055-090, Brazil; Environmental Genomics, Instituto Tecnológico Vale, Belém, Pará 66055-090, Brazil; Environmental Genomics, Instituto Tecnológico Vale, Belém, Pará 66055-090, Brazil; Environmental Genomics, Instituto Tecnológico Vale, Belém, Pará 66055-090, Brazil; Environmental Genomics, Instituto Tecnológico Vale, Belém, Pará 66055-090, Brazil

## Abstract

**Motivation:**

Although high-quality chromosome-scale genome assemblies are feasible, assembling large ones remains complex and resource-intensive. This demands reproducible and automated workflows that not only implement current best practices efficiently but also allow for improvement alongside future updates to those standards.

**Results:**

We present Pipeasm, a Snakemake-based genome assembly pipeline containerized with Singularity. Pipeasm can use HiFi, ONT, and Hi-C data, automating read trimming, nuclear and mitogenome assembly, scaffolding, decontamination, and quality evaluation. Applied to four vertebrate species with distinct genomic characteristics, starting from a single command line and configuration file, it produced assemblies with scaffold L50 proportional to the expected chromosome and genome length, and up to 99.6% BUSCO completeness. Its output also includes detailed reports for each step, genome statistics, Hi-C maps, and files ready for curation.

**Availability and implementation:**

Pipeasm is available at https://github.com/itvgenomics/pipeasm, implemented in Python/Snakemake with Singularity, and runs on Unix-based systems.

## 1 Introduction

Genome sequencing costs are steadily decreasing as the different technologies advance, providing the opportunity to investigate global biodiversity genomics in unprecedented detail. In contrast, the impact of the development of human society started the sixth mass extinction, which threatens most species on the planet (Ceballos *et al.* 2020). Thus, it is fundamental to develop tools not only to accelerate the generation, curation, and analyses of genomic information but also to facilitate the use of such data in conservation efforts, bridging the gap between genomic research and practical conservation actions (Hogg *et al.* 2022, Hogg 2024).

Assessment of life on Earth by genomics has already presented its value for studying evolution [e.g. Feng *et al.* (2020), Zhang *et al.* (2020), Eizirik *et al.* (2023)], conservation [e.g. Formenti *et al.* (2022), Paez *et al.* (2022), Theissinger *et al.* (2023)], and bioeconomy [e.g. Kreplak *et al.* (2019), Zhang et al. (2020), Peng *et al.* (2022)]. In this context, the creation of major initiatives to sequence and assemble Earth’s biodiversity began to take form under umbrella projects such as the Earth Biogenome Project (EBP) (Lewin *et al.* 2018, Gupta 2022). For the assembly of vertebrate genomes, the Vertebrate Genome Project (VGP) has been instrumental in defining the best approaches and metrics, unveiling species-specific characteristics that were previously unattainable (Rhie *et al.* 2021). Brazil’s initiative in this effort was born recently in two projects, the Genomics of the Brazilian Biodiversity (GBB) (Vilaça *et al.* 2024) and the Genotropics (https://www.genotropics.org). Such endeavors have intrinsic difficulties, and the obstacles range from sample collection to the computational infrastructure required to store and analyze the generated data, especially in the global South.

One of the main challenges in generating reference genomes, as highlighted by the EBP, is the assembly and curation of massive genomic datasets on a large scale (Lewin *et al.* 2022). Therefore, it is crucial to develop and maintain modular pipelines to meet deadlines while ensuring adherence to quality standards and requirements (Lawniczak *et al.* 2022), making them accessible with diverse computational resources (Larivière *et al.* 2023). With ongoing advances in data generation and computational hardware, different bioinformatics tools have been developed to automate genome assembly, employing diverse approaches and accommodating various types of sequencing data (Ewels *et al.* 2020, Kuhl *et al.* 2020, Angelova *et al.* 2022, Larivière *et al.* 2023, Obinu *et al.* 2025) (Table 1, available as supplementary data at *Bioinformatics Advances* online). However, continuously evolving and the multi-step assembly procedure can make the assembly process laborious, error-prone, and time-consuming, while also making it difficult to maintain and update workflows built on pipelines that lack flexibility and modularity.

Here, we present Pipeasm, an automated, customizable, and modular genome assembly pipeline designed in line with the best practices proposed in the context of the EBP and VGP to date. Requiring minimal manual intervention, the pipeline covers raw read trimming, overall genome statistics, contig assembly, and automated scaffolding, including genome assembly quality control after every major step, as well as benchmarking and logging. This workflow is built using Snakemake (Köster and Rahmann 2012), a powerful and user-friendly workflow management system, which makes Pipeasm adaptable, transparent, and easy to use in any Linux environment. By using Singularity (Kurtzer *et al.* 2017) containers, Pipeasm ensures that all dependencies are encapsulated, guaranteeing reproducibility and portability across different computing infrastructures. Configuration is streamlined and user-friendly, requiring only editing a single file to set up and customize the workflow, making it accessible to researchers with varying levels of computational expertise. Pipeasm is the main assembly strategy used in the GBB Project and is suggested for vertebrate diploid assemblies of any scale.

## 2 Methods

### 2.1 Pipeline description

**Figure 1:**
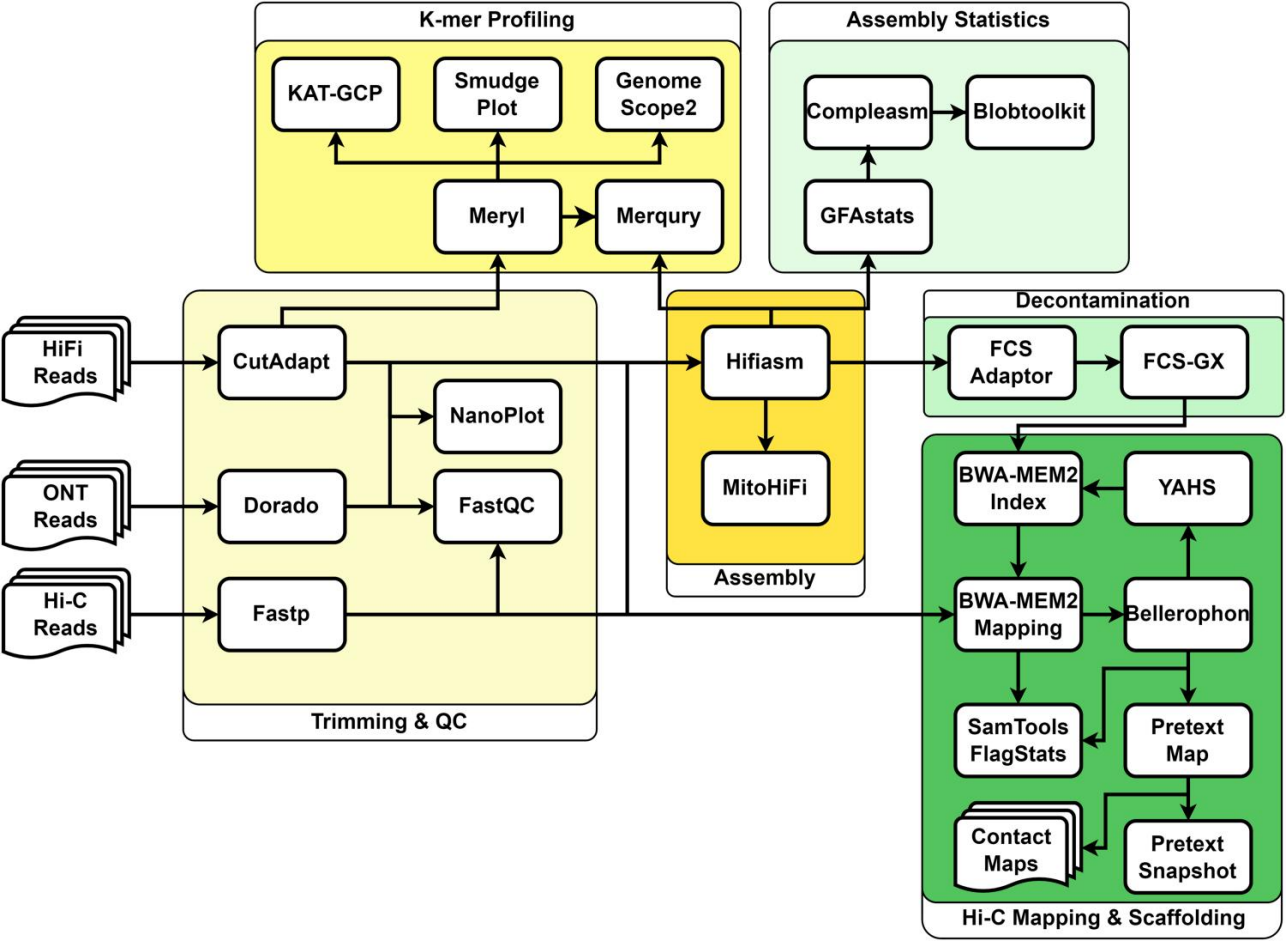
Schematic representation of the Pipeasm workflow, structured into six major tasks (large frames), each executed by the respective presented tools.

Pipeasm integrates a suite of tools to ensure high-quality data, from preprocessing steps to comprehensive reports and statistics. The required input data are PacBio high-fidelity (HiFi-CCS) long reads, which enable solo assembly. In this mode, an assembly is performed using HiFi-only reads, delivering primary and alternate assemblies with partially phased contigs. Optionally, users can provide chromatin-contact (Hi-C) short reads to also perform phased assembly, which uses both HiFi and Hi-C to produce two haplotype-solved assemblies in ideal conditions (Cheng *et al.* 2021). When both data types are available, users can choose to perform only phased assembly for timing optimization or phased and solo assembly. Also, the user can provide Oxford Nanopore Long-Reads (ONT) to perform both solo (along with HiFi reads) and phased (with HiFi and Hi-C reads) assemblies. Pipeasm offers a configuration file (config.yaml) with parameters for all steps, already optimized with default values but easily customizable. The user must provide specific information within this file, including species name, sample ID, reads path, genetic code, taxonomy ID, and the BUSCO database to be used. Pipeasm is organized into six major steps (detailed below): trimming and QC, k-mer profiling, assembly, assembly statistics, decontamination, and Hi-C mapping and scaffolding (Fig. 1).

#### 2.1.1 Trimming and QC

Pipeasm starts trimming PacBio HiFi-CCS long reads and Hi-C short reads using Cutadapt v4.4 (Martin 2011) and Fastp v0.23.4 (Chen *et al.* 2018), respectively, to guarantee high-quality input data for assembly. Dorado v.0.8.1 (https://github.com/nanoporetech/dorado) is used to trim all adapters and primers found in ONT reads. Quality control assessments are performed with FastQC v0.12.1 (https://github.com/s-andrews/FastQC) for both long and short reads, while Nanoplot v1.41.6 (De Coster and Rademakers 2023) is used for long and ultra-long reads.

#### 2.1.2 K-mer profiling

Based on HiFi long reads, Pipeasm performs a set of analyses for detailed genomic statistics. Meryl v1.3 (Rhie *et al.* 2021) is employed for k-mer profiling, including both counting and evaluation. GenomeScope v2.0 (Ranallo-Benavidez *et al.* 2020) estimates genome size, heterozygosity, and repeat content, while SmudgePlot v0.3.0 (Ranallo-Benavidez *et al.* 2020) identifies ploidy levels through K-mer distribution analysis. KAT-GCP v2.4.0 (Mapleson *et al.* 2017) enhances this analysis by providing graphical representations of K-mer spectra, aiding in identifying genomic features and contaminants.

#### 2.1.3 Assembly

The core assembly process is managed by Hifiasm v0.19.6 (Cheng *et al.* 2021), which produces haplotype-resolved genomes and is crucial for removing duplications between haplotypes and ensuring accurate, contiguous assemblies. Pipeasm also applies MitoHifi v3.2.2 (Uliano-Silva *et al.* 2023) to recover the mitogenome, ensuring accurate mitochondrial DNA assembly. GFAstats v1.3.6 (Formenti *et al.* 2021) generates the fasta file from Hifiasm assembled graphs and detailed statistics on the assembled genome, including contig lengths, N50, and scaffold counts.

#### 2.1.4 Decontamination

In post-assembly steps, Pipeasm integrates decontamination algorithms to eliminate remaining contaminants. FCS Adaptor v0.5.0 removes adaptors, while FCS-GX v0.5.0 targets other contaminants (Astashyn *et al.* 2024). As the most recent version of Hifiasm, implemented in Pipeasm, already handles duplication purging, no additional step for this process might be required.

#### 2.1.5 Assembly statistics

The assembly quality control is evaluated through gene-space completeness, assembled K-mers compared to the read’s K-mer distribution, and overall assembly metrics. Compleasm v0.2.2 assesses genome completeness by comparing the assembled genome against BUSCO databases to evaluate the presence of conserved single-copy orthologs (Manni *et al.* 2021, Huang and Li 2023). Merqury v1.3 (Rhie *et al.* 2020) performs the assembled K-mer counts and compares them to the Meryl database created in previous steps. For intuitive visualization, Snailplot, part of BlobToolkit v4.3.5 (Challis *et al.* 2020), provides a comprehensive overview of the assembled genome.

#### 2.1.6 HiC mapping and scaffolding

To elucidate chromosomal interactions, Hi-C reads are mapped onto phased genome assemblies. Genome indexing and mapping are performed using BWA-MEM2 v2.2.1 (Vasimuddin *et al.* 2019) for both haplotypes from decontaminated assemblies. Subsequently, forward and reverse Hi-C reads are aligned separately to each haplotype and sorted with SAMtools v1.19 (Danecek *et al.* 2021). Low-quality alignments and duplicated paired-end reads are filtered and then merged using Bellerophon v1.0 (Hayes and Li 2013), consolidating interaction information into single BAM files for each haplotype.

The quality assessment of the above-mentioned mapping and its merging process is conducted using SAMtools flagstat and the script get_stats.pl (https://github.com/ArimaGenomics/mapping_pipeline), providing essential metrics such as alignment rates, duplication levels, and distance between contact read pairs. Afterwards, the Pretext tools (Map v0.1.9 and Snapshot v0.0.4) (https://github.com/sanger-tol/) convert BAM files into visual representations of Hi-C contact maps. Finally, the automatic scaffolding step, which was performed using YAHS v1.0 (Zhou *et al.* 2023), links contigs into longer scaffolds, enhancing the assembly. The entire mapping step is repeated against the scaffolded genome to generate a final contact map. Then, the last steps of quality check are performed over the scaffolded genome with GFAstats, and genome completeness with Compleasm and Merqury.

#### 2.1.7 Final reports

Pipeasm also compiles multiple metrics and quality assessments from all stages of the genomic assembly process into comprehensive output files. These files encapsulate essential information, such as read quality statistics, genome size estimations, contamination analyses, and assembly quality metrics. The output includes summaries of genome completeness and contiguity, highlighting key indicators such as N50 values, total base pairs, and the presence of conserved genes. Additionally, quality values (QV) and BUSCO scores provide insights into the accuracy and completeness of the assembled genome.

The steps mentioned above are conducted and managed using the Snakemake workflow engine, following its best practices and a standardized repository structure to ensure reproducibility, adaptability, and transparency (Mölder *et al.* 2021). This Python-based approach offers easy readability and automatically scales Pipeasm tasks for parallelization, logging, and benchmarking.

### 2.2 Computational environment and running test

We tested Pipeasm on a cluster with an Intel(R) Xeon(R) Gold 6252 CPU @ 2.10 GHz and 3TB RAM, using 64 threads overall, with 32 threads allocated for each rule within Snakemake. Performance metrics and resource utilization were systematically monitored and extracted using Snakemake’s built-in benchmark tool, ensuring accurate assessment and optimization of computational efficiency throughout the process. It is important to note that the computation time reported is the sum of all CPU time used by all rules rather than the straight running time, providing a comprehensive measure of the workflow’s computational demand.

We employed genomic data from four different species (Table 2, available as supplementary data at *Bioinformatics Advances* online): (i) *Gallus gallus* (bGalGal), a chicken from the family Phasianidae, with a diploid genome size of 1.1 Gbp and 39 chromosomes + WZ (NCBI Genome Accession GCA_027408225.1). Reads were downloaded from the bGalGal5 individual, with 45.88 Gbp PacBio HiFi reads and 147.73 Gbp Arima Hi-C reads. (ii) *Taeniopygia guttata* (bTaeGut), a bird from the family Estrildidae, with a diploid genome size of 1.1 Gbp and 37 chromosomes + WZ (NCBI Genome Accession GCA_009859065.2). Reads downloaded from the bTaeGut2 individual have 39.52 Gbp PacBio HiFi reads and 99.04 Gbp Arima Hi-C reads. (iii) *Elephas maximus* (mEleMax), a mammal from the family Elephantidae, with a diploid genome size of 3.4 Gbp and 27 chromosomes + XY (NCBI Genome Accession GCA_024166365.1). Reads downloaded from the mEleMax1 individual have 137.82 Gbp PacBio HiFi reads and 277.42 Gbp Arima Hi-C reads. (iv) *Pseudophryne corroboree* (aPseCor), a frog from the family Myobatrachidae, with a diploid genome size of 8.9 Gbp and 12 chromosomes (NCBI Genome Accession GCA_028390055.1). Reads downloaded from the aPseCor3 individual have 230.31 Gbp PacBio HiFi reads and 381.02 Gbp Arima Hi-C reads. All above mentioned reads were downloaded from Genome Ark (https://www.genomeark.org).

## 3 Results and discussion

**Figure 2:**
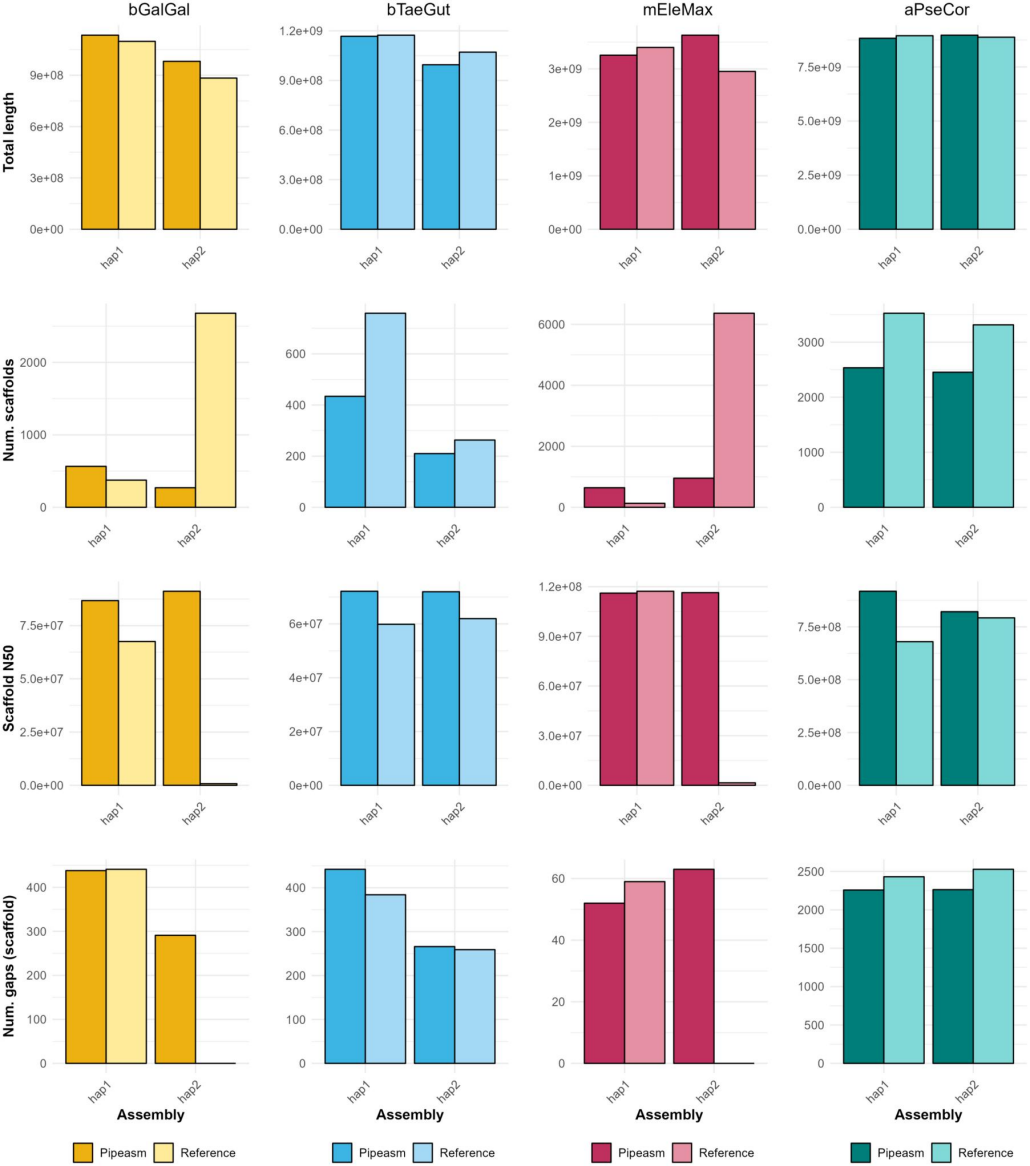
Summary metrics of scaffolding assembly. Key metrics are presented for the references and the results of the Pipeasm scaffolding assembly process. Each row represents a metric, and each column corresponds to a species assembly. Note that the plots’ Y axes are not on the same scale. The first and second haplotypes from different pipelines are not necessarily completely equivalent, as accurate phasing would require trio-based assemblies and further curation. Further, the missing information for reference/original haplotype 2 in mEleMax and bGalGal is likely because these assemblies remain at the contig stage rather than being fully scaffolded.

Pipeasm successfully assembled the data from bGalGal, bTaeGut, mEleMax, and aPseCor (Table 1), reflecting the expected genomic content and similarities with their first assemblies (Fig. 2). Together with k-mer profile analyses (Fig. 1 and Table 3, available as supplementary data at *Bioinformatics Advances* online), the assemblies presented the expected ploidy and range of genome size, as estimated by GoaT, previous assemblies and related species literature (Formenti *et al.* 2021, Rhie *et al.* 2021, Challis *et al.* 2023). In general, haplotypes from the same assembly produced similar statistics, with minor differences in repeat regions and the presence of sex chromosomes, as observed on the contact maps and summary results (Fig. 2, available as supplementary data at *Bioinformatics Advances* online; Table 1). When examining the species genome characteristics, bTaeGut showed the highest heterozygosity (1.34%) and error estimates (0.267), while aPseCor had the largest genome size (8.83 Gb and 8.97 Gb for haplotypes one and two, respectively) and the most repetitive genome (65.5%). Notably, only in mEleMax the number of scaffolds increased from 571 (haplotype 1) and 852 (haplotype 2) in the phased assembly to 642 and 954, respectively, after the scaffolding step, but it also improved overall contiguity. When comparing the Pipeasm assemblies to their respective first-version assemblies, we observed similar or slightly improved statistics (Fig. 2). These reported metrics were obtained from non-curated assemblies, therefore some differences may reflect challenges in assembling each haplotype (primary and alternative), particularly due to repetitive content, leading for example to additional gaps, and the placement of sex chromosomes, which contributed to the larger assembly size. Given that Pipeasm follows the latest best practices from the same consortium that assembled these genomes, differences in algorithms, software versions, and different purging steps may explain the observed improvements. For example, Hifiasm newer updates (e.g. v0.24.0-r702 and 0.23.0-r691) improved assembly quality, contiguity and error corrections (e.g. 0.20.0-r639). Altogether, these results demonstrate that Pipeasm enables the generation of reference-quality assemblies across diverse vertebrate genomes, through a user-friendly framework that generates informative reports and outputs to support subsequent manual curation steps.

**Table 1:** Statistics for genome assembly of *Gallus* (bGalGal), *Taeniopygia guttata* (bTaeGut), *Elephas maximus* (mEleMax), and *Pseudophryne corroboree* (aPseCor), with Hifiasm phased mode followed by YAHS, for both primary (haplotype 1) and alternative (haplotype 2).^a^

Genome stats	bGalGal		bTaeGut		mEleMax		aPseCor	
	Hap1	Hap2	Hap1	Hap2	Hap1	Hap2	Hap1	Hap2
Scaffolds	565	270	434	210	642	954	2,535	2,454
Size length	1.13 Gb	981 Mb	1.17 Gb	995 Mb	3.26 Gb	3.63 Gb	8.83 Gb	8.97 Gb
Largest scaffold	193.55 Mb	197.74 Mb	152.43 Mb	151.85 Mb	241.51 Mb	239.64 Mb	1.746 Gb	1.901 Gb
Scaffold N50	86.73 Mb	91.13 Mb	72.12 Mb	71.94 Mb	116.12 Mb	116.40 Mb	918.47 Mb	821.71 Mb
Scaffold L50	5	4	6	5	10	11	4	4
GC (%)	42.6	42.26	42.92	42.37	41.32	41.55	45.67	45.6
Gaps	438	291	442	266	52	63	2,258	2,263
Single copy (%)	99.57	95.36	99.63	99.91	97.11	99.43	95.35	95.50
Duplicated (%)	0.17	0.14	0.12	0.06	0.31	0.31	0.49	0.55
Fragmented (%)	0.02	0.11	0.06	0.06	0.08	0.03	0.83	0.70
Missing (%)	0.24	4.39	0.18	0.98	2.51	0.24	3.33	3.26
Heterozigosity (%)	1.34		0.25		0.4		0.73	
Read error rate (%)	0.27		0.16		0.11		0.2	
Repeat content (%)	8.5		26.7		65.5		8	

a The main genome assembly metrics were calculated with GFAstats, while completeness was estimated by Compleasm with OrthoDB version 10 verifying: tetrapoda orthologs for aPseCor (5310 genes), aves orthologs for bGalGal (8337 genes), passeriformes orthologs for bTaeGut (10844 genes), and eutheria orthologs for mEleMax (11 636 genes). Heterozigosity, error, and repetitive content estimates were obtained from GenomeScope2.

**Figure 3:**
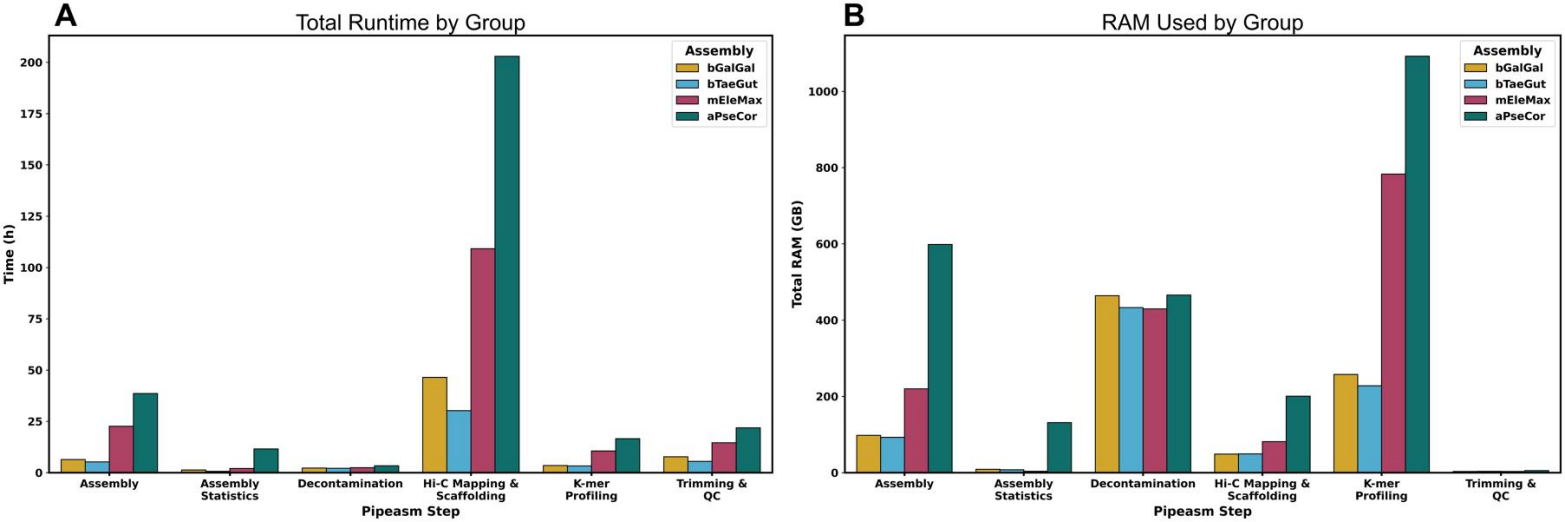
Detailed analysis of running time and RAM usage across various stages of Pipeasm’s combined steps. (A) Running time consumption across different pipeline steps, including Assembly, Assembly Statistics, Decontamination, Hi-C Mapping and Scaffolding, K-mer Profiling, and Trimming and QC. The results are segmented into four different assembly groups: bGalGal, bTaeGut, mEleMax, and aPseCor. The aPseCor group exhibits significantly higher running time, particularly in the Hi-C Mapping and Scaffolding steps. (B) illustrates the total RAM usage for the same pipeline steps and assembly groups. The K-mer Profiling step shows the highest RAM consumption, especially for the aPseCor group, indicating a resource-intensive process.

Benchmarking results for the above assemblies provided insights into resource utilization and computational efficiency across various steps of Pipeasm (Fig. 3). The entire process for only a phased assembly, including scaffolding and Hi-C maps, took approximately 14 h for bTaeGut and approximately 80 h for aPseCol. Running time is mainly affected by genome length, although it is important to note that it also depends on the computational infrastructure. All our assemblies were conducted in a high-performance computing (HPC) environment, and the running times may vary based on factors such as disk input/output operations, CPU clock speed, and memory frequency. Still, the relationship between CPU time and RAM usage will likely remain consistent across different setups. Overall, the observed running time is primarily due to the optimization provided by Snakemake in managing task execution, since Pipeasm: (i) allocates fewer cores to simple tasks that do not serve as prerequisites for others; and (ii) automatically initiates new tasks that can be run concurrently without waiting for previous tasks to be completed or requiring user intervention to start subsequent tasks.

The assembly process was time and memory-intensive, particularly for aPseCor (the largest genome size, with 9 Gb, Table 1), having a significant running time and RAM consumption of approximately 38 h and 600 GB, respectively (Fig. 3). The decontamination step also demanded considerable memory for all species (average of 548 GB RAM), as it used the same FCS-GX database for sequence alignment. The Hi-C mapping and scaffolding stage had an extensive running time (average of 97 h, depending on genome size) but had moderate memory use (average of 94 GB). K-mer profiling had the highest memory consumption (average of 590 GB) but took less time (average of 8.44 h), and adjusting settings in Meryl could help reduce RAM usage. The trimming and quality control steps, though requiring less memory (average of 3.64 GB), still took a notable amount of processing time (average of 12.39 h), especially for aPseCor (21.82 h).

Since Pipeasm requires HiFi reads for assembly, it meets the minimum requirements for achieving high-quality vertebrate genome assemblies. Including Hi-C and/or ONT data and performing phased assembly further elevates the pipeline to higher standards (Rhie *et al.* 2021). Although the scaffolding step is the most time-consuming (Larivière et al. 2023), it is essential for producing genomic information that is ready for manual curation. Moreover, during the execution of the entire pipeline, users can monitor task completion, review output files, and check the analysis logs, ensuring a high degree of transparency (Mölder *et al.* 2021). The evaluation and adjustment of parameters can be achieved without requiring the entire pipeline to be completed before re-running any steps. Tasks that have already been performed are automatically recognized by Snakemake, ensuring that only the necessary steps are re-executed. Indeed, the recent implementation of numerous pipelines for high-throughput sequencing data using Snakemake [e.g. Mölder *et al.* (2021), Mohsen *et al.* (2022), Fallon *et al.* (2023), Gregoricchio and Zwart (2023), Neuenschwander *et al.* (2023), Weber *et al.* (2023)] already underscores its significant value. Finally, given that runtime and memory requirements can vary considerably across genomes, assembly step, and computing environments, implementing these multiple assembly steps within a Snakemake-based pipeline that efficiently manages resources and automates task execution significantly streamlines and simplifies the assembly process for users.

When compared to recently published assembly pipelines, such as SnakeCube (Angelova *et al.* 2022), Chromosome-Scale Assembler (CSA) (Kuhl et al. 2020), and the Galaxy pipeline (Larivière et al. 2023) (Table 1, available as supplementary data at *Bioinformatics Advances* online), Pipeasm presents several differences and advantages. SnakeCube, for example, does not use HiFi or Hi-C data and employs different tools and approaches for assembly, including a polishing step that is less common in current methodologies. CSA also does not use HiFi data and requires an additional re-assembly step. It lacks genome assembly evaluation during the process, and being a Perl script, it may be less transparent, flexible, and readable for some users. While CSA offers good performance in terms of time consumption and the ability to input data from additional species, it employs a methodology that is not widely adopted today.

The Galaxy pipeline adheres to VGP standards and follows a similar concept to Pipeasm. However, Pipeasm’s implementation using Snakemake and Singularity offers greater flexibility and adaptability across diverse computational environments. Its modular architecture allows seamless integration of new tools and workflows, while its clear, readable structure simplifies maintenance and customization. Pipeasm’s transparent design ensures full traceability and reproducibility, empowering users to audit and validate every step of the process. These qualities make Pipeasm not only robust and scalable but also highly accessible for researchers with limited coding experience, without compromising performance or reliability. Although being command-line-based, Pipeasm simplifies execution for non-expert users through a single commented configuration file, abstracting away complex commands and enabling the entire workflow to be launched with a single execution of a bash script. Finally, Pipeasm offers greater scalability than Galaxy for large genome projects that require multiple assemblies. Furthermore, the Galaxy pipeline, whether used online or locally, can be very limiting depending on the region, data, and resources available to the researcher. In contrast, Pipeasm provides a more versatile and accessible solution for various genomic assembly projects.

The Sanger genome assembly tool (https://github.com/sanger-tol/genomeassembly), developed with NextFlow and Colora (Obinu et al. 2025), another pipeline in Snakemake, are also available for genome assembly. However, Pipeasm remains the only one that delivers a genome ready for manual curation, with contact maps generated and a pretext file for scaffold editing. Unlike the others, Pipeasm also provides various k-mer analyses, statistics, and publication-ready figures and reports. Our pipeline stands out for being the most user-friendly, as it does not require manual downloading of any database if you choose not to run the genome decontamination step.

## 4 Conclusions

Pipeasm optimizes analyses and reduces running time compared to executing each step manually, since it automates job scheduling, parallelizes tasks where possible, and minimizes errors in managing multiple scripts, while still maintaining high assembly quality. The vertebrate species used in this study serve as surrogates to the ones to be included in the GBB project, demonstrating that Pipeasm delivers a high-quality genome assembly ready for curation within approximately 1 to 4 straight days, depending on the specific genome structure and available computational resources. The tool also provides a straightforward approach to obtain the most relevant results for evaluating the assembly.

Furthermore, the use of Snakemake endows Pipeasm with valuable readability, modularity, adaptability, and transparency. Users can easily determine which commands will be executed and, with basic coding skills, can add new features according to their needs. Additionally, by utilizing Singularity to containerize the required software, Pipeasm simplifies the provision of all necessary bioinformatics dependencies without installation and facilitates the seamless updating of tools to newer versions or even their replacement with alternative tools as needed.

Pipeasm presents limitations that are intrinsic to the tools used, such as the need for enough computational resources and its restricted applicability to certain organisms. Future development of Pipeasm will focus on implementing trio-based assembly and support for assemblies with more than two haplotypes, further aligning the pipeline with EBP recommendations (Lawniczak *et al.* 2022). Additionally, there is an urgent need for an annotation pipeline, given the challenges observed in reference genome initiative projects (Lewin *et al.* 2022). Currently, Pipeasm significantly facilitates the assembly of vertebrate reference genomes. Its simplicity of use and focus on providing the most useful results directly contribute to the popularization of genomic information. This is a crucial step for large-scale projects focusing on conservation, biodiversity studies, and applications in the bioeconomy.

## Supplementary Material

vbaf326_Supplementary_Data

## Data Availability

Pipeasm is available at https://github.com/itvgenomics/pipeasm. The datasets used in the assemblies and the generated results can be found at https://zenodo.org/records/17243106.

## References

[vbaf326-B1] Angelova N , Danis T, Lagnel J et al SnakeCube: containerized and automated pipeline for de novo genome assembly in HPC environments. BMC Res Notes 2022;15:98. 35255960 10.1186/s13104-022-05978-5 PMC8900408

[vbaf326-B2] Astashyn A , Tvedte ES, Sweeney D et al Rapid and sensitive detection of genome contamination at scale with FCS-GX. Genome Biol 2024;25:60. 38409096 10.1186/s13059-024-03198-7 PMC10898089

[vbaf326-B3] Ceballos G , Ehrlich PR, Raven PH et al Vertebrates on the brink as indicators of biological annihilation and the sixth mass extinction. Proc Natl Acad Sci USA 2020;117:13596–602. 32482862 10.1073/pnas.1922686117 PMC7306750

[vbaf326-B4] Challis R , Richards E, Rajan J et al BlobToolKit—interactive quality assessment of genome assemblies. G3 (Bethesda) 2020;10:1361–74. 32071071 10.1534/g3.119.400908 PMC7144090

[vbaf326-B5] Challis R , Kumar S, Sotero-Caio C et al Genomes on a tree (GoaT): a versatile, scalable search engine for genomic and sequencing project metadata across the eukaryotic tree of life. Wellcome Open Res 2023;8:24. 36864925 10.12688/wellcomeopenres.18658.1 PMC9971660

[vbaf326-B6] Chen S , Zhou Y, Chen Y et al fastp: an ultra-fast all-in-one FASTQ preprocessor. Bioinformatics 2018;34:i884–90. 30423086 10.1093/bioinformatics/bty560 PMC6129281

[vbaf326-B7] Cheng H , Concepcion GT, Feng X et al Haplotype-resolved de novo assembly using phased assembly graphs with hifiasm. Nat Methods 2021;18:170–5. 33526886 10.1038/s41592-020-01056-5 PMC7961889

[vbaf326-B8] Danecek P , Bonfield JK, Liddle J et al Twelve years of SAMtools and BCFtools. Gigascience 2021;10:giab008. 33590861 10.1093/gigascience/giab008 PMC7931819

[vbaf326-B9] De Coster W , Rademakers R. NanoPack2: population-scale evaluation of long-read sequencing data. Bioinformatics 2023;39:btad311. 37171891 10.1093/bioinformatics/btad311 PMC10196664

[vbaf326-B10] Eizirik E , de Ferran V, Sartor CC, et al Conservation genomics of neotropical carnivores. In: Galetti PM (ed.), Conservation Genetics in the Neotropics. Cham: Springer International Publishing, 2023, 475–501.

[vbaf326-B11] Ewels PA , Peltzer A, Fillinger S et al The nf-core framework for community-curated bioinformatics pipelines. Nat Biotechnol 2020;38:276–8. 32055031 10.1038/s41587-020-0439-x

[vbaf326-B12] Fallon TR , Čalounová T, Mokrejš M et al transXpress: a snakemake pipeline for streamlined de novo transcriptome assembly and annotation. BMC Bioinformatics 2023;24:133. 37016291 10.1186/s12859-023-05254-8 PMC10074830

[vbaf326-B13] Feng S , Stiller J, Deng Y et al Dense sampling of bird diversity increases power of comparative genomics. Nature 2020;587:252–7. 33177665 10.1038/s41586-020-2873-9 PMC7759463

[vbaf326-B14] Formenti G , Rhie A, Balacco J et al; Vertebrate Genomes Project Consortium. Complete vertebrate mitogenomes reveal widespread repeats and gene duplications. Genome Biol 2021;22:120. 33910595 10.1186/s13059-021-02336-9 PMC8082918

[vbaf326-B15] Formenti G , Theissinger K, Fernandes C et al; European Reference Genome Atlas (ERGA) Consortium. The era of reference genomes in conservation genomics. Trends Ecol Evol 2022;37:197–202. 35086739 10.1016/j.tree.2021.11.008 PMC13065249

[vbaf326-B16] Gregoricchio S , Zwart W. snHiC: a complete and simplified snakemake pipeline for grouped Hi-C data analysis. Bioinform Adv 2023;3:vbad080. 37397353 10.1093/bioadv/vbad080 PMC10307938

[vbaf326-B17] Gupta PK. Earth biogenome project: present status and future plans. Trends Genet 2022;38:811–20. 35599020 10.1016/j.tig.2022.04.008

[vbaf326-B18] Hayes M , Li J. Bellerophon: a hybrid method for detecting interchromo-somal rearrangements at base pair resolution using next-generation sequencing data. BMC Bioinformatics 2013;14:S6–9. 10.1186/1471-2105-14-S5-S6 PMC3622635 23734783

[vbaf326-B19] Hogg CJ. Translating genomic advances into biodiversity conservation. Nat Rev Genet 2024;25:362–73. 38012268 10.1038/s41576-023-00671-0

[vbaf326-B20] Hogg CJ , Ottewell K, Latch P et al Threatened species initiative: empowering conservation action using genomic resources. Proc Natl Acad Sci USA 2022;119:e2115643118. 35042806 10.1073/pnas.2115643118 PMC8795520

[vbaf326-B21] Huang N , Li H. Compleasm: a faster and more accurate reimplementation of BUSCO. Bioinformatics 2023;39:btad595. 37758247 10.1093/bioinformatics/btad595 PMC10558035

[vbaf326-B22] Kreplak J , Madoui M-A, Cápal P et al A reference genome for pea provides insight into legume genome evolution. Nat Genet 2019;51:1411–22. 31477930 10.1038/s41588-019-0480-1

[vbaf326-B23] Kuhl H , Li L, Wuertz S et al CSA: a high-throughput chromosome-scale assembly pipeline for vertebrate genomes. Gigascience 2020;9:giaa034. 32449778 10.1093/gigascience/giaa034 PMC7247394

[vbaf326-B24] Kurtzer GM , Sochat V, Bauer MW et al Singularity: scientific containers for mobility of compute. PLoS One 2017;12:e0177459. 28494014 10.1371/journal.pone.0177459 PMC5426675

[vbaf326-B25] Köster J , Rahmann S. Snakemake—a scalable bioinformatics workflow engine. Bioinformatics 2012;28:2520–2. 22908215 10.1093/bioinformatics/bts480

[vbaf326-B26] Larivière D , Abueg L, Brajuka N et al Scalable, accessible, and reproducible reference genome assembly and evaluation in galaxy. *Nat Biotechnol* 2024;42:367–70. 10.1038/s41587-023-02100-3 PMC11462542 38278971

[vbaf326-B27] Lawniczak MKN , Durbin R, Flicek P et al Standards recommendations for the earth BioGenome project. Proc Natl Acad Sci USA 2022;119:e2115639118. 35042802 10.1073/pnas.2115639118 PMC8795494

[vbaf326-B28] Lewin HA , Robinson GE, Kress WJ et al Earth BioGenome project: sequencing life for the future of life. Proc Natl Acad Sci USA 2018;115:4325–33. 29686065 10.1073/pnas.1720115115 PMC5924910

[vbaf326-B29] Lewin HA , Richards S, Lieberman Aiden E et al The earth BioGenome project 2020: starting the clock. Proc Natl Acad Sci USA 2022;119:e2115635118. 35042800 10.1073/pnas.2115635118 PMC8795548

[vbaf326-B30] Manni M , Berkeley MR, Seppey M et al BUSCO: assessing genomic data quality and beyond. Curr Protoc 2021;1:e323. 34936221 10.1002/cpz1.323

[vbaf326-B31] Mapleson D , Garcia Accinelli G, Kettleborough G et al KAT: a K-mer analysis toolkit to quality control NGS datasets and genome assemblies. Bioinformatics 2017;33:574–6. 27797770 10.1093/bioinformatics/btw663 PMC5408915

[vbaf326-B32] Martin M. Cutadapt removes adapter sequences from high-throughput sequencing reads. EMBnet J 2011;17:10–2.

[vbaf326-B33] Mohsen A , Chen Y-A, Allendes Osorio RS et al Snaq: a dynamic snakemake pipeline for microbiome data analysis with QIIME2. Front Bioinform 2022;2:893933. 36304319 10.3389/fbinf.2022.893933 PMC9580898

[vbaf326-B34] Mölder F , Jablonski KP, Letcher B et al Sustainable data analysis with snakemake. F1000Res 2021;10:33. 34035898 10.12688/f1000research.29032.1 PMC8114187

[vbaf326-B35] Neuenschwander S , Cruz Dávalos DI, Anchieri L et al Mapache: a flexible pipeline to map ancient DNA. Bioinformatics 2023;39:btad028. 36637197 10.1093/bioinformatics/btad028 PMC9901408

[vbaf326-B36] Obinu L , Booth T, De Weerd H et al Colora: a Snakemake workflow for complete chromosome-scale de novo genome assembly. 2025;41:btaf175. 10.1093/bioinformatics/btaf175 PMC12065627 40238183

[vbaf326-B37] Paez S , Kraus RHS, Shapiro B et al; Vertebrate Genomes Project Conservation Group. Reference genomes for conservation. Science (1979) 2022;377:364–6. 10.1126/science.abm8127 35862547

[vbaf326-B38] Peng Y , Yan H, Guo L et al Reference genome assemblies reveal the origin and evolution of allohexaploid oat. Nat Genet 2022;54:1248–58. 35851189 10.1038/s41588-022-01127-7 PMC9355876

[vbaf326-B39] Ranallo-Benavidez TR , Jaron KS, Schatz MC et al GenomeScope 2.0 and smudgeplot for reference-free profiling of polyploid genomes. Nat Commun 2020;11:1432. 32188846 10.1038/s41467-020-14998-3 PMC7080791

[vbaf326-B40] Rhie A , Walenz BP, Koren S et al Merqury: reference-free quality, completeness, and phasing assessment for genome assemblies. Genome Biol 2020;21:245. 32928274 10.1186/s13059-020-02134-9 PMC7488777

[vbaf326-B41] Rhie A , McCarthy SA, Fedrigo O et al Towards complete and error-free genome assemblies of all vertebrate species. Nature 2021;592:737–46. 33911273 10.1038/s41586-021-03451-0 PMC8081667

[vbaf326-B42] Theissinger K , Fernandes C, Formenti G et al; European Reference Genome Atlas Consortium. How genomics can help biodiversity conservation. Trends Genet 2023;39:545–59. 36801111 10.1016/j.tig.2023.01.005

[vbaf326-B43] Uliano-Silva M , Ferreira JGRN, Krasheninnikova K et al; Darwin Tree of Life Consortium. MitoHiFi: a python pipeline for mitochondrial genome assembly from PacBio high fidelity reads. BMC Bioinformatics 2023;24:288. 37464285 10.1186/s12859-023-05385-y PMC10354987

[vbaf326-B44] Vasimuddin M , Misra S, Li H. Efficient architecture-aware acceleration of BWA-MEM for multicore systems. In: 2019 IEEE International Parallel and Distributed Processing Symposium (IPDPS). Rio de Janeiro, Brazil: IEEE, 2019, 314–24.

[vbaf326-B45] Vilaça ST , Vidal AF, D’Oliveira Pavan AC et al Leveraging genomes to support conservation and bioeconomy policies in a megadiverse country. Cell Genom 2024;4:10068. 10.1016/j.xgen.2024.100678 PMC11605686 39423822

[vbaf326-B46] Weber T , Cosenza MR, Korbel J et al MosaiCatcher v2: a single-cell structural variations detection and analysis reference framework based on strand-seq. Bioinformatics 2023;39:btad633. 37851409 10.1093/bioinformatics/btad633 PMC10628386

[vbaf326-B47] Zhang Q-J , Li W, Li K et al The chromosome-level reference genome of tea tree unveils recent bursts of non-autonomous LTR retrotransposons in driving genome size evolution. Mol Plant 2020;13:935–8. 32353626 10.1016/j.molp.2020.04.009

[vbaf326-B48] Zhou C , McCarthy SA, Durbin R et al YaHS: yet another Hi-c scaffolding tool. Bioinformatics 2023;39:btac808. 36525368 10.1093/bioinformatics/btac808 PMC9848053

