# Supplementary material for: Pipeasm: a tool for automated large chromosome-scale genome assembly and evaluation
Source: Bioinform Adv. 2026 Jan 2;6(1):vbaf326. doi: 10.1093/bioadv/vbaf326 (PMC12800776; doi:10.1093/bioadv/vbaf326)
Supplement: vbaf326_Supplementary_Data [file vbaf326_supplementary_data.pdf]

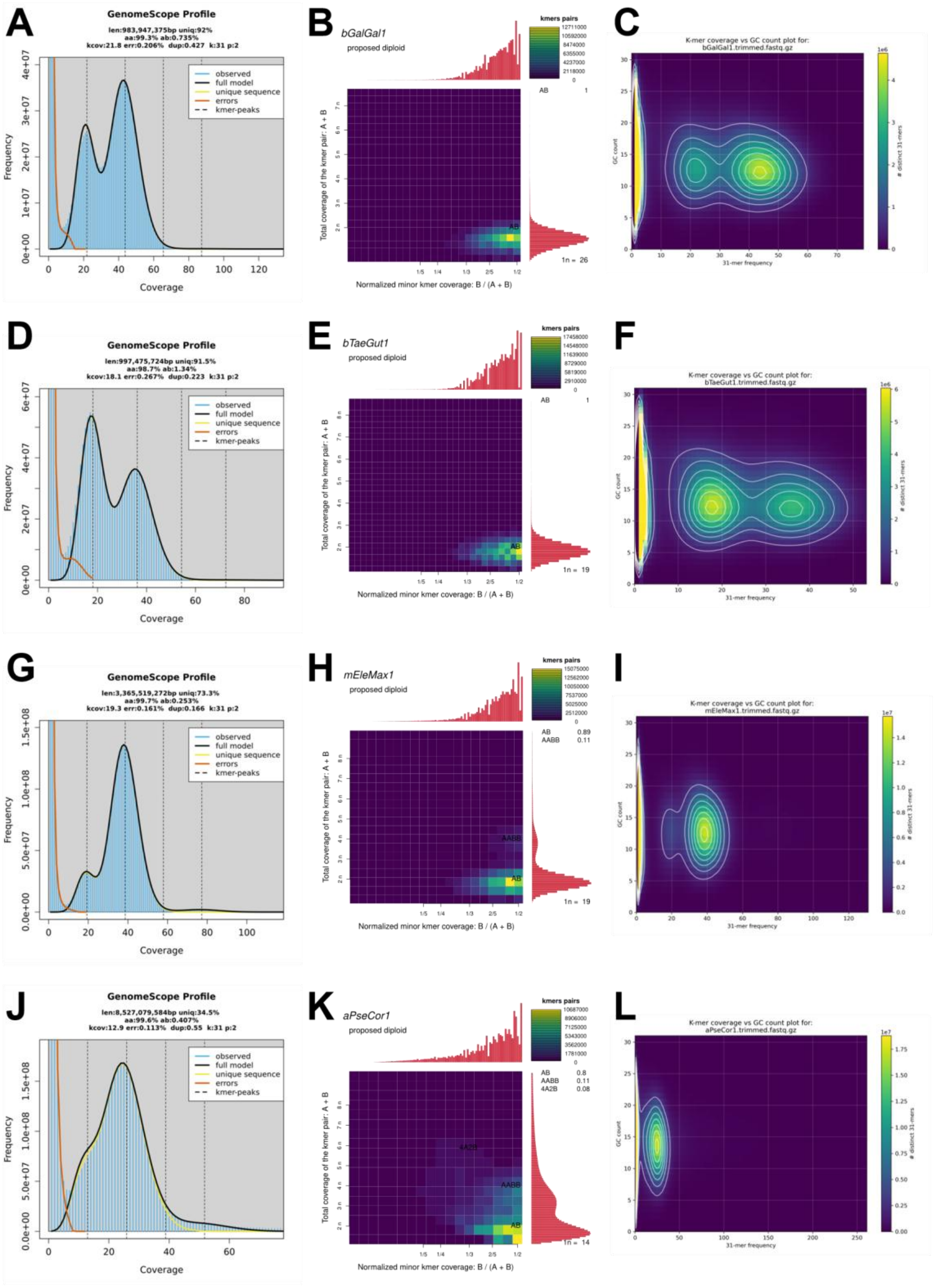

Figure S1. Summary metrics of species genomes based on k-mer distribution. Panels A-C stand for bGalGal, D-F for bTaeGut, G-I for mEleMax and J-L for aPseCor. Left panels are GenomeScope2 results, showing predicted genome length, duplication rate, heterozygosity. Middle panels are SmudgePlot results, showing estimated ploidy and k-mer depth. The right panels are KAT-GCP results showing k-mer frequency per GC count. All estimates presented used k=31.

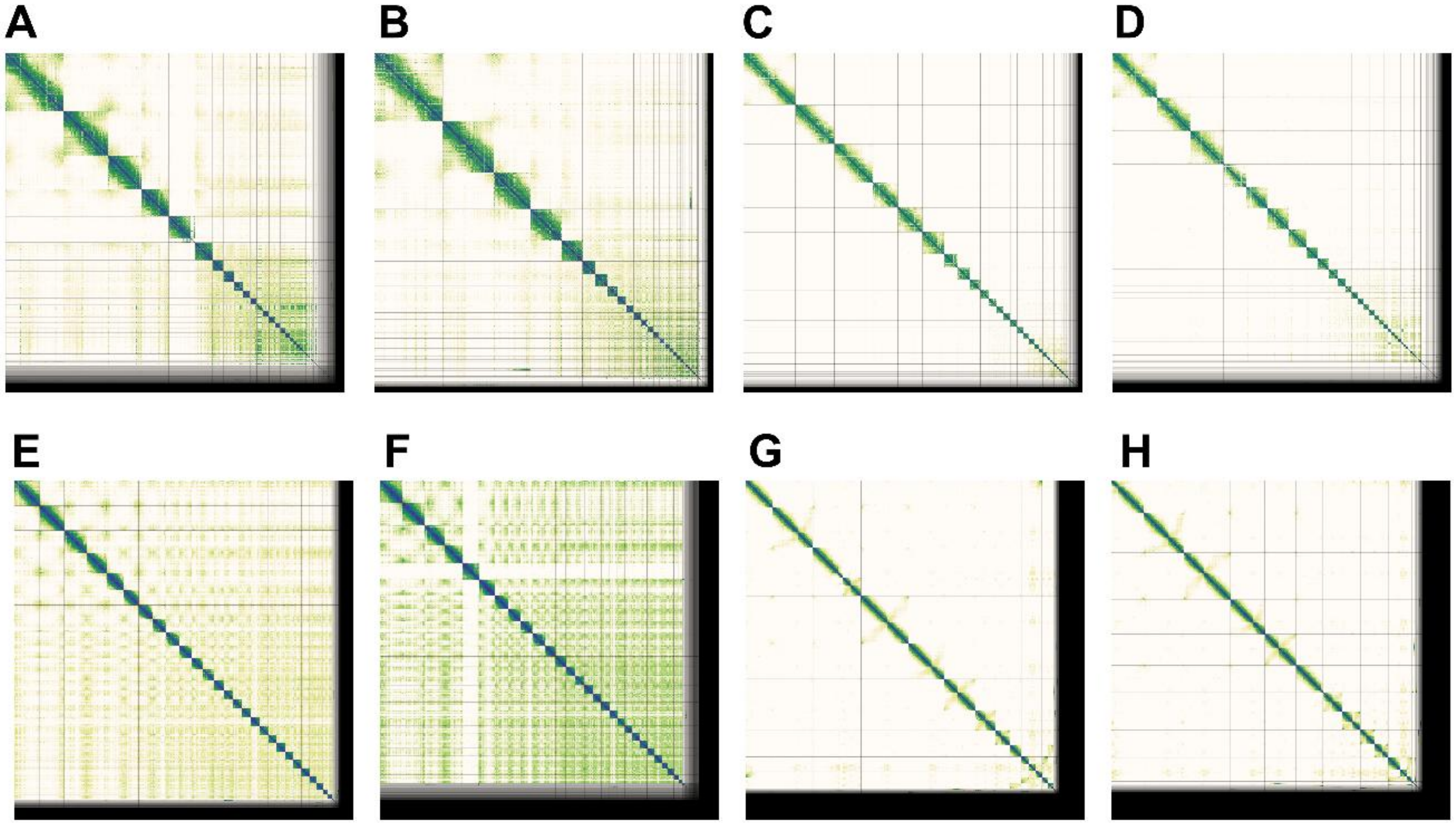

Figure S2. Pretext snapshots of both haplotype 1 and haplotype 2 of the bGalGal (A, B), bTaeGut (C, D), mEleMax (E, F), and aPseCor (G, H) assemblies, respectively.

Pipeline	Softwares						
	**Orchestrator**	**Trimming & QC**	**k-mer Profiling**	**Assembly**	**Assembly Statistics**	**Decontamination**	**Hi- C mapping & Scaffolding**
Pipeasm	Snakemake	CutAdapt, Dorado, Fastp, NanoPlot, FastQC	KAT-GCP, SmudgePlot, GenomeScope2, Meryl, Merqury	Hifiasm	GFAstats, Compleasm, Blobtoolkit	FCS-Adaptor, FCS-GX	Arima Genomics Mapping Pipeline, YAHS, Pretext, Samtools Stats
Galaxy-VGP	Galaxy's workflow engine	CutAdapt, MultiQC	Meryl, Mequry, GenomeScope2	Hifiasm	GFAstats, BUSCO, Compleasm	-	-
Sanger-ToL	Nextflow	-	FastK k-mer database already run, GenomeScope2, Merqury.FK	Hifiasm	GFAstats, BUSCO	Purge_dups	BWA-MEM2/Minimap2, YAHS
Colora	Snakemake	Fastp, NanoPlot	KMC, GenomeScope2	Hifiasm	GFAstats, QUAST, BUSCO	FCS-Adaptor, FCS-GX, Purge_dups	Arima Genomics Mapping Pipeline, YAHS
CSA	Perl	-	-	WTDBG2	-	-	-
SnakeCube	Snakemake	NanoPlot, FastQC	-	Flye	QUAST, BUSCO	-	-

Table S1: Comparative overview of genome assembly pipelines and tools. Summaries of genome assembly pipelines, their orchestrators (workflow managers), and the key software used at each stage of the assembly process. The stages include raw read trimming and quality control (QC), k-mer profiling, assembly, assembly statistics, decontamination, and Hi-C mapping with scaffolding. It shows commonly used tools and workflow systems (Snakemake, Nextflow, Galaxy) implemented in Pipeasm, Galaxy-VGP [19], Sanger-ToL [20,54], Colora [23], CSA [21], and SnakeCube [22]

Species (code)	Family	Genome size (Gbp)	Chromosomes	Accession	PacBio HiFi data (Gbp)	Hi-C data (Gbp)
Gallus gallus (bGalGal)	*Phasianidae*	1.1	39 + WZ	GCA_027408225.1	45.88	147.73
Taeniopygia guttata (bTaeGut)	*Estrildidae*	1.1	37 + WZ	GCA_009859065.2	39.52	99.04
Elephas maximus (mEleMax)	*Elephantidae*	3.4	27 + XY	GCA_024166365.1	137.82	277.42
Pseudophryne corroboree (aPseCor)	*Myobatrachidae*	8.9	12	GCA_028390055.1	230.31	381.02

Table S2: Genomic datasets used for Pipeasm evaluation. Summary of the four species used to test the pipeline, including genome size, chromosome count, NCBI accession, and the amount of PacBio HiFi and Hi-C sequencing data retrieved from Genome Ark (https://www.genomeark.org).

Species	GenomeScope2									SmudgePlot	
	Predicted Length (MB)	uniq (%)	aa (%)	ab (%)	kcov	err (%)	dup	k-mer	ploidy	1n	Proposed Ploidy
bGalGal	983,947	92	99.3	0.735	21.8	0.206	0.427	31	2	26	Diploid
bTaeGut	997,475	91.5	98.7	1.34	18.1	0.267	0.223	31	2	19	Diploid
mEleMax	3,365,519	73.3	99.7	0.253	19.3	0.161	0.166	31	2	19	Diploid
aPseCor	8,527,079	34.5	99.6	0.407	12.9	0.113	0.55	31	2	14	Diploid

Table S3. GenomeScope2 and SmudgePlot metrics summarizing genome size, heterozygosity, k-mer composition, and ploidy inference for bGalGal, bTaeGut, mEleMax and aPseCor based on 31-mer analyses.
